# The Interactions Between Cancer Stem Cells and the Innate Interferon Signaling Pathway

**DOI:** 10.3389/fimmu.2020.00526

**Published:** 2020-03-31

**Authors:** Laura Martin-Hijano, Bruno Sainz

**Affiliations:** ^1^Cancer Stem Cell and Tumor Microenvironment Group, Department of Biochemistry, Universidad Autónoma de Madrid (UAM), Madrid, Spain; ^2^Cancer Stem Cell and Tumor Microenvironment Group, Department of Cancer Biology, Instituto de Investigaciones Biomédicas “Alberto Sols” (IIBM), CSIC-UAM, Madrid, Spain; ^3^Cancer Stem Cell and Tumor Microenvironment Group, Chronic Diseases and Cancer—Area 3, Instituto Ramón y Cajal de Investigación Sanitaria (IRYCIS), Madrid, Spain

**Keywords:** interferons, cancer stem cells, immune response, plasticity, immunoediting, quiescence

## Abstract

Interferons (IFNs) form a family of cytokines with pleiotropic effects that modulate the immune response against multiple challenges like viral infections, autoimmune diseases, and cancer. While numerous anti-tumor activities have been described for IFNs, IFNs have also been associated with tumor growth and progression. The effect of IFNs on apoptosis, angiogenesis, tumor cell immunogenicity, and modulation of immune cells have been largely studied; however, less is known about their specific effects on cancer stem cells (CSCs). CSCs constitute a subpopulation of tumor cells endowed with stem-like properties including self-renewal, chemoresistance, tumorigenic capacity, and quiescence. This rare and unique subpopulation of cells is believed to be responsible for tumor maintenance, metastatic spread, and relapse. Thus, this review aims to summarize and discuss the current knowledge of the anti- and pro-CSCs effects of IFNs and also to highlight the need for further research on the interplay between IFNs and CSCs. Importantly, understanding this interplay will surely help to exploit the anti-tumor effects of IFNs, specifically those that target CSCs.

## Introduction

### Interferons

Interferons (IFNs) constitute a family of cytokines first described in the late 1950s for their ability to trigger a very potent anti-viral response in cells (1). All IFNs are class II α-helical cytokines that are classified into three main types: IFN-I (mainly IFN-α and -β) (2), IFN-II (IFN-γ) (3), and IFN-III (IFN-λ) (4) and their canonical signaling consists of activation of the JAK/STAT pathway (5).

IFNs are fundamental players in the modulation of both innate and adaptive immune responses. Although they were first identified as molecules with a strong capability of inducing viral resistance in cells, many other activities have been discovered for this family of cytokines over the years, including their involvement in pathologies such as autoimmune diseases [e.g., systemic lupus erythematosus (6–9) and rheumatoid arthritis (8, 10, 11)] and cancer (discussed below). IFNs, regardless of the specific receptor they activate, are able to exert pleiotropic effects, suggesting a rich signaling network coupled to IFN stimulation and undoubtedly adds complexity to understanding its effects on cell function and its contributions to immune response regulation.

#### Type I Interferon (IFN-I)

**Figure 1:**
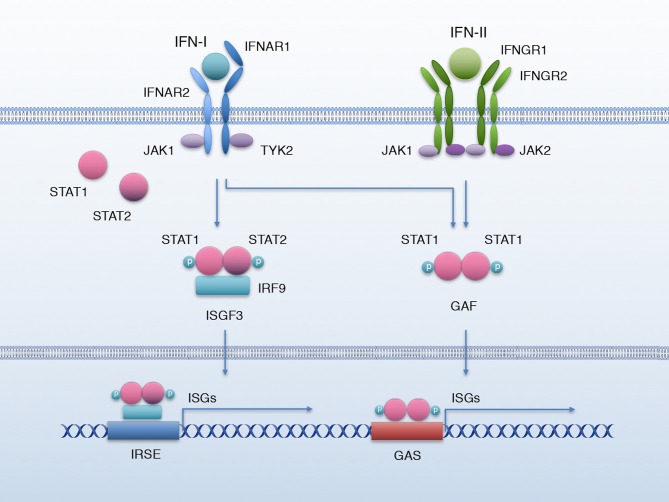
IFN-I and IFN-II signaling. Canonical IFN-I and IFN-II signaling pathway. Binding of IFN-I to IFNAR activates JAK1 and TYK2 to phosphorylate STAT1 and STAT2, which associate with IRF9 to form the transcriptional complex ISGF3; ISGF3 translocates to the nucleus to bind the IRSE elements and activate the transcription of a battery of ISGs. IFN-II biding to its receptor IFNGR activates kinases JAK1 and JAK2, which phosphorylate STAT1; p-STAT1 forms a homodimer named GAF that translocates to the nucleus and activates the transcription of ISGs by binding GAS elements. IFN-I can also lead to the formation of GAF.

IFN-I comprises multiple and diverse members; in mammals, 9 subtypes have been described: IFN-α (of which there are 13 known subtypes), IFN-β, -ε, -κ, -ω, -δ, -τ, -v, and -ζ; all of them except -δ and -τ exist in humans (12). The level of homology between these members can range from 20% to nearly 100% (2). However, they all signal through the same receptor, the IFN-α receptor (IFNAR). IFN-I binds a heterodimeric receptor formed by IFNAR1 and IFNAR2 chains, causing their constitutively associated Janus kinases TYK2 and JAK1, respectively, to activate and phosphorylate signal transducer and activator of transcription 1 (STAT1) and 2 (STAT2). pY-STAT1 and pY-STAT2 then form a heterodimer that associates IRF9 to form a transcriptional activator complex named IFN-stimulated gene factor 3 (ISGF3). ISGF3 translocates to the nucleus, where it binds interferon-stimulated response elements (IRSE) to activate the transcription of a battery of interferon-stimulated genes (ISGs) (Figure 1). However, IFN-I also activates other non-canonical signaling pathways such as the MAPK (13, 14), PI3-Kinase (15–17), and NF-κB pathways (18, 19), as well as unphosphorylated STAT1 (U-STAT1) (5), that prolongs the expression of a subset of interferon-induced immune regulatory genes (20).

Regarding their immunomodulatory nature, IFN-I fulfills roles in both innate and adaptive immune responses, that include inducing cell-autonomous antiviral activity (21), stimulating immune cells, including natural killer (NK) (22–24) and T cells (25–29), and increasing antigen presentation by macrophages and dendritic cells (30), in order to help orchestrate an efficient immune response (5).

#### Type II Interferon (IFN-II)

IFN-II has only one member, IFN-γ, which is remarkably different from IFN-I in structure and has a different receptor, but was originally grouped in the IFN family due to its ability to trigger an antiviral response (31). Like other IFNs, IFN-γ also activates the JAK/STAT signaling pathway, through IFNGR. This receptor is formed by two chains: IFNGR1 and IFNGR2. Binding of IFN-γ to its receptor activates the associated Janus kinases JAK1 and JAK2, respectively to IFNGR-1 and−2, to phosphorylate STAT1. pY-STAT1 forms homodimers, also known as interferon gamma-activated factor (GAF), that translocate to the nucleus to activate the transcription of a set of ISGs by binding the interferon-activated sites (GAS) (5) (Figure 1). Nonetheless, like IFN-I, IFN-γ can also activate other non-canonical signaling pathways such as MAPK (32, 33), PI3-Kinase (32, 33), and NF-κB (34, 35) and U-STAT1 (5, 20).

Functionally, IFN-γ also importantly contributes to the development of innate and adaptive immune responses, targeting mainly macrophages (36–39) and T cells (40–42). IFN-γ signaling induces the expression of many transcription factors, that can amplify the IFN response (5). Importantly, IFN-γ has a substantial role in modulating macrophage activation, as it upregulates the expression of gene products with microbicidal activity (43–46) and interacts with other cytokines and signaling molecules to enhance or antagonize their effect (47–50). Also, IFN-γ is capable of modulating helper T cell (Th) responses (51–53) and promoting class switching in B cells (54, 55). In addition, IFN-γ modulates the activity and recruitment of NK cells (56, 57). Interestingly, IFN-γ has been reported to either promote or repress NK cell-mediated lysis of tumor cells derived from diverse pediatric tumor cell lines (58). Treatment of the tumor cell lines with IFN-γ induced differential upregulation of MHC-class I and ICAM-I, which seemed to determine tumor cells' resistance or sensitivity, respectively, to NK cell-mediated lysis.

#### Type III Interferon (IFN-III)

Finally, interferon type III or IFN-λ is the latest class to be described, and it also shares the same antiviral functions as that of type I IFNs (2). The focus of this review will be on the effect that type I and type II IFNs have on cancer stem cells (CSCs) in different cancer entities.

### Interferons in Cancer

Decades of research have demonstrated that IFNs are able to display a wide range of anti-tumor activities, including induction of apoptosis, inhibition of angiogenesis and proliferation, cell terminal differentiation and immune regulation. At the level of tumor cell survival, IFNs can induce tumor cell apoptosis through various mechanisms, such as the TRAIL pathway (59, 60), via CD95/Fas (61, 62) and the activation of pro-apoptotic members of the Bcl-2 family [reviewed by Kotredes and Gamero (63)]. Likewise, IFNs can impede tumor expansion by inducing cell cycle arrest. IFNs can up- or down-regulate CDK inhibitors and c-Myc expression, respectively, to inflict an anti-proliferative effect on tumor cells, amongst other mechanisms (64–67). However, IFNs have other indirect forms of fighting tumors, such as inducing oxygen and nutrients supply deprivation of tumor cells by suppressing angiogenesis, thus creating a hypoxic and acidic microenvironment. IFNs are also able to elicit inhibition of angiogenesis by downregulating the expression of potent angiogenic factors in endothelial and stromal cells, including IL-8, platelet-derived growth factor (PDGF) and vascular endothelial growth factor (VEGF), and in tumor cells, such as fibroblast growth factors (FGFs) (68–72). Furthermore, angiogenesis inhibition can result from IFN-mediated impairment of proliferation and migration of endothelial cells (70, 71, 73, 74).

Importantly, as already mentioned, IFNs are key regulators of the immune response against tumors. IFN-α, -β, and -γ are able to directly upregulate the expression of surface tumor-associated antigens (75, 76) via augmentation of MHC I class and MHC II class molecules (77), thus increasing the immunogenicity of tumor cells and making them more vulnerable to identification and subsequent destruction by the immune system. Indirect/unspecific immunoregulatory effects of IFNs encompass activation of dendritic cells to cross-present tumor antigens to T cells (78), promotion of full CD8^+^ maturation necessary for them to elicit their cytotoxic effects (29, 79, 80), prevention of the proliferation of T regulatory cells, as well as enhancement of T helper cell function (81, 82) and promotion of macrophage polarization toward an M1-like pro-inflammatory state instead of the M2 pro-tumoral state (83), thus eluding their immunosuppressive effect (83, 84), amongst other mechanisms (85).

Alternatively, pro-tumoral properties have also been described for IFNs. While classically considered as pro-apoptotic agents, it has been shown that IFN-α/β activate the NF-κB pathway, inducing cell survival and protecting tumor cells against apoptotic stimuli in a variety of cancer types (86, 87). Also, IFNs can upregulate survival factors that protect cells against apoptotic stimuli, including MCL1, increased in myeloma presumably via STAT3 (88), and G1P3, which has been reported to promote tumor cell survival and contribute to poor outcome of patients in estrogen-receptor positive breast cancer (89). IFNs can also act as proliferative stimuli (90). For example, IFITM1 is an IFN-induced protein whose expression was shown to enhance lung cancer cell proliferation *in vitro* and tumor growth *in vivo* (91). In addition, IFITM1 expression was reported to promote invasion in head and neck cancer (92). Interestingly, IFN-α has been reported to induce endothelial cell proliferation, thus fomenting angiogenesis (93). One of the most recognized pro-tumoral activities of IFNs is the induction or overexpression of a subset of ISGs in distinct cancers, identified as an IFN-related DNA damage-resistant signature (IRDS), that confers tumor cell resistance to therapy (94, 95). Also, high expression of IRDS genes has been shown to promote tumor growth and metastasis (92, 96). Another major role of IFNs in cancer is immunomodulation and, in this regard, IFNs have been shown to promote immunoevasion via upregulation of the expression of MHC I class molecules, thus decreasing sensitivity to NK cells (97), downregulation of tumor-associated antigen presentation (98, 99), upregulation of the cytotoxic T cell inhibitor PDL-1 in tumor cells (100, 101), and promotion of a tumorigenic TME milieu (102).

### Interferons as Anticancer Therapy

Intensive research focused on IFNs' anti-tumor activities finally led to the approval of IFN-α by the FDA as the first cancer immunotherapy in 1986 (103). In spite of being discovered for their anti-viral activities, IFN-α2a and IFN-α2b have been used as anticancer therapeutic agents across multiple cancer types, including hairy cell leukemia, chronic myelogenous leukemia (CML) (104), AIDS-related Kaposi's sarcoma, follicular lymphoma, multiple myeloma, melanoma, condyloma acuminate, hepatocellular carcinoma (HCC), and cervical intraepithelial neoplasms (105, 106). IFN-β use as an anticancer drug is still under study, although ongoing phase III trials for melanoma (107, 108) and for glioma (109) and glioblastoma (110) are being conducted with promising results. However, IFN-β treatment studies in metastatic breast cancer have not been as successful (111). IFN-γ has also been explored as a therapy for cancer, showing some contrasting results. While IFN-γ treatment has proved to increase survival in ovarian cancer (112) and prevent recurrence in bladder cancer (113), it did not achieve the same results in other malignancies such as melanoma (114), leukemia (115), colorectal (116), and pancreatic cancers (117). Unfavorably, other preclinical studies have shown how IFN-γ upregulation leads to increased metastasis in melanoma (97) and breast cancer (118). It is worth noting that IFN treatment presents adverse side-effects ranging from a flu-like syndrome consisting of fever, chills, headache, myalgia, arthralgia, anorexia and fatigue (119), to neuropsychiatric symptoms, being depression a frequent disorder with a prevalence of 30–70% (120). These adverse effects are dose-limiting and may lead to treatment cessation in severe or longstanding treatment cases.

### CSC Model

Cancer stem cells (CSCs) constitute a subpopulation of tumor cells endowed with stem-like properties such as tumorigenesis, metastatic dissemination potential, chemoresistance, and relapse (121). Nowadays, the most accepted CSC model proposes that, on the one hand, CSCs remain in a de-differentiated state, maintain their pluripotency and have unlimited self-renewal capacity. However, on the other hand, they can also differentiate into all possible cancer cell states that form a continuum, thus building the tumor hierarchy and giving rise to intratumoral heterogeneity (122). These unique abilities define CSCs as the sole drivers of tumorigenesis and tumor maintenance, and subsequently the cell entity that drives metastatic spread.

CSCs are pluripotent due to the reactivation of embryonal signaling pathways, such as Sonic Hedgehog (SHH), WNT, NOTCH, and Bone Morphogenetic Protein (BMP) (123). Other classical pluripotent genes expressed by these cells include *KLF4* (124), *NANOG* (125), *OCT3/4* (125, 126), *SOX2* (125–127), and the NODAL/ACTIVIN axis (128). CSCs are also characterized by the expression of stem-like markers, some of which are associated with a cancer type and some of which are more broadly expressed. Some of the most commonly used stem-like markers to identify CSCs are CD24, CD44, CD133, ALDH1, and CXCR4 (129–131). However, not every CSC express the same stem-like markers, the latter being due to the heterogeneity that exists within the CSC population. Genetically and/or epigenetically diverse CSC subpopulations possess different characteristics, that allow them to or preclude them from adapting to challenging situations such as nutrient deprivation, hypoxia, chemotherapy, or immune pressure. This unequal fitness of each CSC subpopulation drives either their clonal expansion or retreat (121), thus driving tumor evolution.

CSCs maintenance is supported by specific niches within the tumor. Importantly, interaction with the TME is crucial for CSC niche formation. A dynamic communication and influence occur between CSCs and the TME, thus assembling a balanced loop of reciprocal modeling. Of note, CSCs represent only a small percentage of the total number of tumor cells, but they are dispersedly located within different CSC niches and present distinct phenotypes. While some niches are spatially distinct (e.g., hypoxic and perivascular regions), others are defined by cellular interactions (e.g., immune niche). These tumor ecology dynamics have been elegantly described and reviewed by Prager et al. (132).

Implicit in this model is the idea/concept of CSC plasticity. Classically, the cellular populations with the ability to differentiate or transition into different lineages (e.g., hematopoietic stem cells) possess phenotypic plasticity. However, we now know this is not a unidirectional process, and that progenitor, transient and differentiated cells are able to regain stem-like properties that drives them to a pluripotent state (121). The latter is greatly influenced by stem cell niche factors. In this same way, CSC niches provide the needed signals for more differentiated cells to activate their plasticity and go back to a stem-like state if necessary. From a clinical perspective, not only CSC targeting but also CSC niche elimination would be necessary for complete cancer eradication.

EMT is a crucial process for activating plasticity and stemness. Between the pure epithelial (E) and pure mesenchymal (M) states, there is a spectrum of intermediate conditions, being the hybrid E/M state, with both epithelial and mesenchymal features, the state that represents the population of cells with the highest plasticity and stemness, increased therapy resistance, tumor-initiating capacity and metastatic potential (133). Importantly, these E/M hybrids with stem-like properties are able to form clusters, with increased apoptosis-resistance, that enter into the bloodstream where they collectively migrate to distant sites and colonize them more successfully than pure mesenchymal-state motile tumor cells (134, 135). As part of CSC plasticity, these cells are also able to enter a state of reversible quiescence, that is actively maintained. Quiescence protects CSCs from cell-cycle targeted therapies and grants them long-term survival through activation of environmental stress adaptive responses, including metabolic reprogramming and mechanisms that favor genomic integrity protection. In addition, these cells present high tumorigenic potential (136). Quiescent CSCs can exist within the tumor, as a subpopulation that does not contribute to tumor growth but that is greatly resistant to adverse conditions and can reactivate and re-enter the cell cycle when in the presence of certain ques or when more favorable conditions are achieved (137). They can also appear as disseminated dormant tumor cells, that are maintained in a non-proliferating state for long periods of time and can reactivate driving relapse and metastasis (136).

Chemoresistance is another hallmark of CSCs (138, 139). CSCs are invulnerable to conventional anticancer therapies, as they have an intrinsic chemo- and radio-resistant profile that enables their survival and clonal expansion over those cells unable to resist therapeutic pressures. Expression of drug efflux pumps, such as ABCG2 and MDR1, not only allows CSCs to evade the lethal impact of chemotherapy (140), but these pumps also seem to promote stem-like capacities via facilitating the clearance of endogenous anti-tumorigenic molecules from the cell while redirecting pro-tumorigenic molecules to the cell's surface receptors (141). Other common mechanisms of chemoresistance are ALDH activity, expression of pro-survival BCL-2 protein family members and activation of several signaling pathways involved in chemo- and radio-resistance, including MYC and AKT1 (142).

Without a doubt, CSCs represent a population of highly complex tumor cells with unique properties that are responsible for tumor progression, chemoresistance, dormancy, and metastasis. At the same time, these cells are divided into unique subpopulations whose nature is driven/influenced by CSC niches, thus promoting different phenotypes (i.e., plasticity). With this in mind, the only way to successfully eradicate cancer would be to eliminate CSCs and simultaneously target the cues that promote CSC maintenance/plasticity or the source of those cues (i.e., CSCs niches).

It is well-known that IFNs can exert many different anti-tumor effects that negatively affect tumor viability, but less is known about their specific impact on CSCs. In fact, the few studies that have tested the relationship between IFNs and CSCs have yielded opposite and contradictory conclusions, showing both pro and anti-tumor activity. Thus, this review will try to set the story straight by discussing this complex relationship and provided data to support both sides.

## Stemness and Tumorigenic Potential

### Interferon Type I

A recent study by Castiello et al. (143) showed how IFNAR1 silencing had a significant impact on the CSC subset in an HER2/neu transgenic mouse model (neuT) of breast cancer. Loss of functional IFNAR1 not only resulted in earlier onset and increased tumor multiplicity, but also in the presentation of a gene expression profile associated with aggressive human breast cancer. In line with these results, IFNAR^−/−^ tumors showed an enrichment in the ALDH1^+^ CSC compartment, which demonstrated a greater self-renewal capacity *in vitro* and tumorigenic potential *in vivo*. These results clearly propose IFN-I as a negative regulator of stemness in breast cancer tumor cells. Accordingly, Doherty et al. (144) obtained similar conclusions when studying the role of IFN-β on triple-negative breast cancer (TNBC) CSCs, using an *in vitro* model of primary human mammary epithelial cells (HMEC) virally transduced with transforming factors. Within transformed cells, a subpopulation of mesenchymal-like cells with CSCs properties emerged (Mes/CSC), while the remaining cells maintained an epithelial phenotype and did not present such properties (Ep/non-CSC). Regarding IFN signaling, Mes/CSCs presented a basal repression of numerous ISGs, while Ep/non-CSCs had an IFN gene expression signature. Inhibition of ISG expression was attributed to upregulation of unphosphorylated ISG3F (U-ISG3F) in Mes/CSCs, which is part of the alternative IFN-I signaling pathway, although the origin of its activation remains unclear. In order to test whether IFN-β was able to reactivate the canonical IFN pathway, Mes/CSCs and Ep/non-CSCs were treated with IFN-β and CSCs properties were tested *in vitro*, showing a reversion of the CSC status of Mes/CSC cells. Moreover, IFN-β reactivated the expression of ISGs in Mes/CSCs by upregulating P-ISG3F. Therefore, activation of the canonical IFN-I pathway by IFN-β inhibited the stem-like capacities of Mes/CSCs in this model.

In support of this, Yuki et al. (145) had previously reported IFN-β to reduce proliferation, self-renewal capacity, and tumorigenesis in human glioma-initiating cells (GICs) by inducing their terminal differentiation into oligodendrocytes via STAT3 activation. Treatment of patient tumor-derived cells with IFN-β induced the phosphorylation and subsequent activation of STAT3, leading to a cell-cycle arrest in G0/G1, decreased clonogenic capacity, reduction of the expression of stem markers and, importantly, terminal differentiation of the GICs into oligodendrocytes. Significantly, STAT3 had been previously linked to gliogenesis by Bonni et al. (146) and Rajan and McKay (147), who described how Ciliary Neurotrophic Factor (CNTF)-mediated activation of STAT3 promoted the differentiation of cortical precursor cells and multipotent stem cells of the central nervous system, respectively, into astrocytes. More recently, STAT3 activation has been linked to regulation of human neural stem cell differentiation (148) and to promotion of the differentiation of NG2 cells (oligodendrocytes progenitors) into oligodendrocytes after a contusive spinal cord injury (149). Likewise, STAT3 has been shown to mediate IL-6-induced neuroendocrine differentiation in prostate cancer cells (150). This pro-differentiating role for STAT3 contradicts previous work describing its role in promoting CSCs traits among different cancer types (151–155), thus highlighting the importance of the tumor context. Illustrating this complex regulation, another study underscored the role of IFN-I as a repressor of glioblastoma stem-like cells (GSCs), as it appeared to inhibit the proliferation and self-renewal capacity of GSCs. However, the authors claim that IFN-I also inhibits the ability of GSCs to differentiate into astrocytes, since it only induces a transient activation of STAT3, while induction of astrocytic differentiation results from sustained activation of STAT3 (156).

Another interesting study tested the effects of IFN-β produced intracellularly on lung cancer murine cells (LL), avoiding external treatment of cells with the recombinant cytokine (157). For that purpose, LL cells were transduced with the mouse *ifn-b* (rBV/IFN-β) gene using a baculovirus vector (BV) and subjected to several tumor-specific assays. rBV/IFN-β cells showed a lower proliferation rate and, importantly, decreased anchorage-independent growth (i.e., CSC self-renewal), compared to control cells. Consistent with these results, a reduction in the tumorigenic and metastatic capacity of rBV/IFN-β cells was observed, strengthening the link between IFN-β and inhibition of stem-like capacities.

IFN-α has also been reported to specifically target the side population (SP) of ovarian cancer cells, a subset of cells endowed with stem-like properties (i.e., CSCs) (158). In an attempt to exploit the anti-tumor effects of IFN-α, ovarian PDXs were subjected to gene therapy with IFN-α, and results showed a marked increase in survival rate in those PDXs bearing a high proportion of SP cells compared to those containing a low proportion, indicating that IFN-α specifically and negatively affects the CSC compartment. Accordingly, treatment of isolated SP cells with IFN-α resulted in decreased proliferation and self-renewal capacity of these cells and in a dramatic change in their transcriptional profile, compared to non-SP cells. Moreover, these findings were tested in CRC and Daoy medulloblastoma cells with similar results, indicating that this negative regulation of the CSC compartment could be extended to other cancer types.

In contrast to the CSC inhibitory role of IFN-I described above, other studies have come to different conclusions. For example, Ma et al. (159) revealed that IFN-α fostered stem-like properties in oral squamous cell carcinoma (OSCC) cells. Treatment of implanted tumor xenografts with IFN-α resulted in increased expression of stemness markers and tumor growth. Similar results at the level of stemness markers and increased self-renewal capacity were also observed *in vitro* with OSCC cells treated with IFN-α.

More recently, a robust link between death receptor CD95/Fas, IFN-I-dependent activation of STAT1 and stemness in different cancer types has been described by Qadir et al. (160). CD95 is an apoptosis-inducing death receptor, although it can also participate in a variety of tumor promoting activities. In fact, chronic stimulation of CD95 in tumor cells has been reported to increase the number of CSCs in breast cancer (161). In this work, the authors observed that long-term stimulation of CD95 in tumor cells led to type IFN-I production and secretion, and subsequent activation of the IFN-I pathway. In MCF-7 breast cancer cells, activation of the IFN-I pathway resulted in increased expression of stem-like markers. Moreover, cell sorting of MCF-7 breast cancer cells using the stem marker CD44 revealed that CD44^+^ cells had higher levels of STAT1 expression than CD44^−^ cells. In addition, treatment with IFNα/β induced/increased ALDH1 activity and self-renewal capacity. To further confirm the role of IFN-I as a driver of stemness, IFN-β pre-treated cells were used in a limiting dilution assay (LDA), which revealed the ability of IFN-β to enhance tumorigenic potential *in vivo*. These findings are not limited to one cancer type, as the authors were able to show similar results for GBM and squamous cell carcinoma (SSC). Interestingly, knocking-down STAT1 resulted in abrogation of STAT2 and STAT3 phosphorylation, concomitant with a loss of IFN-I-induced stem-like properties, suggesting the involvement of STAT2 and STAT3 activation in mediating the observed CSCs promoting effects of IFN-I in a STAT1-dependent manner. Overall, this thorough study strongly suggests IFN-I as a cancer stemness driver in breast cancer, SCC and GBM, involving activation of STAT1, STAT2, and STAT3.

In line with this, IFN-β has also been linked to tumor stemness promotion in pancreatic ductal adenocarcinoma (PDAC). Sainz et al. (162) described an intimate communication between tumor-associated macrophages (TAMs) and pancreatic CSCs in primary tumor tissues and derived cultures. Interestingly, PDAC cells polarized resident TAMs toward an M2 phenotype, which in turn actively secreted high levels of ISG15, an interferon-stimulated gene. ISG15 can act as a free molecule—intracellularly or in the tumor milieu—and it can also conjugate to proteins as a ubiquitin-like modifier through a process known as ISGylation (163). In this work, TAM-secreted ISG15 was found to enhance the stem-like properties of PDAC CSCs *in vivo* and *in vitro*, promoting their self-renewal, tumorigenic, chemoresistant and migratory capacities, in addition to higher levels of intracellular ISGylation, which have also been related to CSC promotion in nasopharyngeal carcinoma (164). Strikingly, TAMs secreted ISG15 in response to IFN-β secretion by pancreatic CSCs, thus establishing an intricate communication between CSCs and TAMs that resulted in reinforcement of stem-like properties in pancreatic CSCs. The fact that tumor cells (or CSCs) can secrete IFNs is not a novel concept. In 2011 Tsai et al. (165) described that ZR-75-1 breast cancer cells secreted elevated levels of IFN-β, which in turn contributed to Ras transformation. In addition, sarcoma, melanoma and leukemia tumor cells have been described to secrete IFN-α in response to doxorubicin treatment (166). Moreover, inflammatory breast cancer (IBC) cells have been reported to secrete high levels of IFN-α to the TME milieu, which contributed to increase its pro-tumorigenic character (102, 167). In addition to an IFN-α-secreting phenotype, IBC cells showed an upregulation of the IFN-α signaling pathway. Interestingly, Monsurrò et al. (168) identified two molecular phenotypes of PDAC based on differential expression of ISGs; the “anti-viral state” phenotype was characterized by increased resistance to oncolytic viral infection and was associated with activation of hypoxia pathways and increase of HLA proteins expression.

### Interferon Type II

Regarding IFN-II, a study by Ni et al. (169) investigated the impact of IFN-γ on a specific subpopulation of quiescent colon CSCs (i.e., Label-retaining cancer cells or LRCCs), isolated from primary colon tumors based on PKH26/67 high staining. This work revealed that IFN-γ selectively targeted LRCCs due to their overexpression of IFNGR, compared to non-LRCCs. The authors showed that IFN-γ treatment of LRCCs greatly inhibited their self-renewal and tumorigenic capacities and induced apoptosis, while non-LRCCs were less affected. Therefore, in this context and in this model system, IFN-γ was proposed as a selective anti-CSC agent.

Another relevant study by Song et al. (170) explored the connection between endogenous IFN-γ levels and tumor stemness in a cohort of non-small cell lung cancer (NSCLC), esophageal squamous cell carcinoma (ESCC), CRC and HCC patients. Strikingly, the study revealed that low-IFN-γ levels in tumor interstitial fluid (TIF) strongly correlated with poor prognosis, TNM tumor staging, brain metastasis and chemoresistance. In line with this, NSCLC, ESCC, CRC, and HCC patients with low TIF-IFN-γ levels showed higher CD133 and Vimentin expression, as well as increased tumor stemness-related and EMT-related gene expression. *In vitro* treatment of NSCLC cell lines with high and low doses of IFN-γ revealed that low dose treatments increased the self-renewal capacity and expression of stem-like makers. In line with this observation, *in vivo* treatment of NSCLC-derived cell lines with a low IFN-γ dose resulted in higher frequency of CD133^+^ cells compared with those treated with a high IFN-γ dose, indicating an enrichment in the CSC compartment due to low IFN-γ stimulation. Dissection of the signaling cascade behind these effects revealed that low IFN-γ treatment of NSCLC cell lines induced I-CAM expression, which activated the PI3K-Akt-Notch1 axis leading to increased stemness. On the other hand, high IFN-γ doses induced apoptosis via the JAK1/STAT1/caspases pathway. This work not only illustrates the complex regulation of IFN signaling, but it also describes the opposing effects that can be achieved with the very same molecule using different dosing strategies. In addition, the results of this study are likely applicable to the immunoediting process, in which infiltrated effector T-cells and NK cells initially produce high levels of IFN-γ in the TME, resulting in tumor cell apoptosis. However, this initial immune response wave can eventually lead to T-cell and NK cell exhaustion and dysfunctional activity (171, 172), thus decreasing IFN-γ production and generating an IFN-γ-low TME with tumor stemness promoting capacity. Importantly, such a scenario could also occur during the earlier stages of tumor development in so-called “cold tumors” that are poorly infiltrated with immune cells. Interestingly, however, this study may also help to resolve the contradictory pro- and anti-tumor effects described above for both IFN-I and IFN-II, which may be the result of the doses of IFNs used across the different studies.

Very recently, Matteucci et al. (173) described and reviewed the pivotal role of human endogenous retrovirus (HERVs) activation in the promotion and maintenance of pluripotency and stem-like properties in melanoma CSCs. The authors also highlight the correlation between HERVs activation and aggressiveness features across several types of cancer. In this line, in the same year Cañadas et al. (174) described a very interesting interplay between IFN-γ and a particular subtype of HERVs named Stimulated 3 prime antisense retroviral coding sequences (SPARCS), which are located in the 3′ untranslated region of IFN-γ-inducible genes. Strikingly, IFN-γ induces the activation of SPARCS-containing genes—many of which are involved in innate immune regulation—resulting in the promotion of a more aggressive mesenchymal-like state of SCLC cells and in the production of cytosolic dsRNA through the bi-directional transcription of target genes. In turn, dsRNA can be sensed via the RIG-I/MAVS or the cGAS/STING pathways, which induce the production of IFNs, thus creating a positive feedback loop. Of note, IFN-γ induced the overexpression of PD-L1, which correlated with high baseline expression of the stem-like marker CD44. Moreover, deletion of *MAVS* significantly reduced the tumorigenic capacity of SCLC tumor cells. In summary, this work highlights the role of IFN-γ in activating the transcription of SPARCS and its impact on SCLC cells phenotype and opens the door to considering IFN-γ-induced SPARCS activation as a regulator of stem-like features in SCLC tumor cells.

## Invasion, Migration, and Metastasis

### Interferon Type I

In ovarian cancer, Li et al. observed that IFI27, an IFN-α inducible protein, was upregulated in patient tumor tissue samples, compared to their paired healthy controls, and correlated with poor disease-free survival. The authors subsequently found IFI27 to not only be a driver of stemness (175), but this IFN-induced protein could also promote EMT, resulting in increased migration and invasion. It is well-known that EMT is one of the driving biological processes of stemness in tumor cells (176, 177), and in this work the authors make a very unique connection between EMT induction by an IFN-α stimulated gene and acquisition of stem-like properties such as increased self-renewal and drug resistance. In accordance with this observation, Zhu et al. (178) also described IFN-α as a promoter of stemness in PDAC. In an attempt to unveil possible differences of the effects of IFN-α on CSCs and non-CSCs, two PDAC cell lines with opposing stem markers levels were used: MiaPaca (low levels) and Panc1 (high levels). The authors showed in their study how IFN-α treatment of both PDAC cell lines reduced cell viability and proliferation *in vitro*, while simultaneously increasing the expression of CSCs cell surface markers, suggesting IFN-α induces a CSC enrichment, likely via killing off non-CSCs. In order to confirm these results *in vivo*, an orthotopic PDAC mouse model was used. While administration of IFN-α to mice reduced tumor volume in comparison to the control group, CSCs markers were significantly upregulated, suggesting again an enrichment in CSCs. Along these lines, IFN-α-treated mice presented more colon metastases compared to the non-IFN-treated control group. In summary, these results suggest that IFN-α treatment of PDAC cells leads to elimination of the tumor bulk cells resulting in an enrichment of the CSC compartment, concomitant with a boost in metastatic spread. However, based on the concept of plasticity, it is also feasible that non-CSCs converted into CSCs, contributing to the enrichment of the CSC population.

### Interferon Type II

In head and neck squamous cell carcinomas (HNSCC), as in many other cancer types, the CXCL12/CXCR4 axis is involved in metastatic dissemination (179). As metastasis formation is one of the hallmarks of CSCs, CXCR4 is often used as a stem-like marker for the identification of CSCs with enhanced metastatic capacity (180). In this respect, Katayama et al. (181) performed a study to determine the effects of IFN-γ on CXCR4 expression and function in several HNSCC cell lines. Histological analysis of primary tumors and metastases from a cohort of 56 patients revealed high levels of CXCR4 in tumor cells, but not in healthy head and neck tissue, which correlated with poor prognosis. In addition, CXCL12 expression was barely detectable in the primary tumor stromal tissue, but was strongly expressed in metastatic lymph node stroma, illustrating the CXCR4/CXCL12 axis as a highly plausible mechanism for metastatic spread in this cancer. In this study, the authors aimed to regulate CXCR4 levels in HNSCC cell lines using IFN-γ as an inhibitor, since IFN-γ had been previously shown to downregulate expression of CXCR4 in immune cells like neutrophils (182). Interestingly, they discovered that IFN-γ treatment induced a downregulation of CXCR4, and this downregulation translated into an inhibition in the migratory and invasive capacities of HNSCC cells, as well as CXCR4/CXCL12 axis-mediated cell proliferation. Thus, these authors proposed IFN-γ as a modulator of CXCR4 functional expression and as an inhibitor of HNSCC cell migration induced by this receptor.

Interestingly, during the late 1980's, multiple studies explored the relationship between IFN-γ and metastasis in mice. Firstly, Taniguchi et al. (97) observed that treatment of H-2-deficient non-metastatic B16 melanoma cells with physiological doses of IFN-γ (1–10 U/ml) was sufficient to decrease cell growth *in vitro* and, surprisingly, to increase the lung-colonizing potential of these cells *in vivo*. Treatment with IFN-β was also able to induce the same metastatic phenotype, although a 1,000-fold higher concentration was required to observe similar effects. Investigating the mechanisms behind this IFN-γ-mediated or -enhanced metastasis, it appeared that IFN-γ induced a higher expression of surface H-2, that enabled tumor cells to resist NK-mediated killing. Other studies published in the very same year supported the main concept of Taniguchi et al.'s. work but in melanoma and colon cancer (183–186). More recent studies have also reported the capacity of IFN-γ to promote invasion and metastasis (187), and to act as a double-edge sword in cancer (188, 189). These results suggest that local endogenous IFN-γ released in the TME may play a pivotal role in modulating tumor cells' sensitivity to innate and adaptive immune cells and therefore in their capacity to colonize other organs and metastasize. Again, as shown by Song et al. above, the concentration of IFN-γ at a specific given time during the evolution of the tumor may be critical for IFN-γ to act as a pro- or anti-metastatic/invasive factor.

## Dormancy

### Interferon Type I

It is generally recognized that tumor cell dormancy represents a major obstacle when it comes to effectively treating cancer, as dormant cells are more chemoresistant and upon treatment cessation, these cells can drive tumor relapse. In a recent study, Liu et al. (190) dissected the impact of IFN-β in melanoma CSCs, establishing a previously unknown association with dormancy. In this work, murine and human implanted tumors in mice were treated *in vivo* with IFN-β. Subsequent analysis of isolated single tumor cells revealed that IFN-β treated tumors had a higher proportion of G_0_/G_1_ cells, which were not senescent. In fact, sorting cells using the CSC cell surface marker CD133 revealed that while IFN-β treatment did not reduced the CD133^+^ CSC compartment, IFN-β did induce cell cycle arrest in CD133^+^ and not in CD133^−^ cells, suggesting a specific effect of IFN-β on CSCs. Interestingly, both murine and human CD133^+^ “tumor repopulating cell” (TRC)-derived tumors showed halted growth when treated with IFN-β and a quick re-growth after IFN-β withdrawal, indicating that IFN-β induces a reversible dormancy in melanoma cells. Further studies *in vitro* supported these findings. Specifically, IFN-β treatment of CD133^+^ murine and human melanoma cells in soft 3D fibrin gels induced G_0_/G_1_ cell cycle arrest, expression of dormancy markers, decreased glucose consumption and higher resistance to chemotherapy, many of these features being hallmarks of CSCs. Consistently, IFN-β was not able to induce dormancy in 2D-cultured cells, which are conditions that favor cell differentiation over CSC enrichment. Moreover, knocking-down either STAT1 or STAT2 abolished the IFN-β-mediated quiescence induction in melanoma cells, confirming IFN-β as the driver of dormancy in these cells. Finally, a thorough study of the signaling pathway responsible for this effect underlined the IDO/Kyn/AhR cascade and serine-phosphorylation of STAT3 as the effectors, providing new insights into tumor dormancy mechanisms associated with IFNs.

### Interferon Type II

A similar approach to the Liu et al. (190) study was conducted by the same group using IFN-γ (191), and similar results at the level of stemness promotion were obtained in murine TRCs (i.e., stem cell-like cancer cells that can repopulate tumors). Again, the authors showed that IFN-γ treatment resulted in IDO1/AhR-dependent p27 induction, that prevented STAT1 signaling, suppressing cell death and inducing tumor cell-dormancy in murine TRCs. Importantly, a similar effect with IFN-γ was also shown in human melanoma, breast cancer and HCC cell lines, again through the IDO/AhR/p27 pathway. While Liu et al. dissected the molecular signaling pathway behind IFN-γ-mediated tumor cell-dormancy, Farrar et al. (192) discovered in 1999 that IFN-γ produced by CD8^+^ T cells played a major role in inducing tumor cell dormancy *in vivo*; however, the authors did not dissect the mechanism of action. In their study, a model of tumor dormancy was used, in which a murine B cell lymphoma (BCL_1_) implanted in immunocompetent mice previously immunized with the BCL_1_-derived Ig to orchestrate an anti-Id immune response could be induced into a dormant state. Adoptive transfer of Id-immune CD8^+^ T cells into SCID mice administered with α-BCL_1_-Ig, concomitant with α-IFN-γ antibodies, resulted in complete abrogation of the induction and maintenance of tumor dormancy. These results indicated that endogenous production of IFN-γ by CD8^+^ T cells, in collaboration with humoral immunity, induced and maintained tumor cell dormancy *in vivo*. In line with this, Kmieciak et al. (193) reported 4 years later that CD8^+^ T cell-produced IFN-γ was able to induce apoptosis in those tumor cells expressing high levels of IFNGR, while those expressing low levels entered into a quiescent state. In addition, relapsed tumor-cells presented increased expression of cell surface stem-like markers and higher tumorigenic capacity *in vivo*, thus connecting IFN-γ stimulation in a subset of tumor cells with a quiescent phenotype and a subsequent enrichment in the CSC compartment after tumor regrowth.

## Discussion

The regulation of IFN signaling has been extensively investigated, and yet there are still many aspects that are not fully understood and many questions remain unresolved. An example is the question of how IFN-α and -β are able to exert different effects on cells while signaling through the same receptor—IFNAR—via the JAK/STAT pathway. We now know that IFN stimulation and subsequent downstream effects are highly dependent on the cell type, IFN dose and the cell surface-receptor density in the stimulated cell. Likewise, factors behind the regulation of IFN receptor presentation and IFN secretion levels are numerous and vary (2). This scenario highlights the importance of the cellular and environmental context in which a cell is stimulated by IFNs, and CSCs are no exception. Thus, more research is needed to fully characterize and dissect the factors that mediate the different responses of distinct CSCs to IFNs, described in this review. While we have put forth several possible explanations, including IFN dosing, more studies are still needed. Nevertheless, it is highly likely that what we will discover are cell-type specific effects. For example, regarding IFNs and dormancy, it is known that IFN-α is able to activate dormant hematopoietic stem cells (HSCs), inducing them to proliferate and making them more vulnerable to anti-cycling therapies such as 5-fluorouracil (194); however, while CSCs share many common features with normal stem cells, they also possess an aberrant malignant behavior based in part on a very different signaling circuitry. Thus, the very same stimulus can have completely different effects on normal- and cancer- stem cells. This is certainly the case with respect to the dormancy-specific studies described in this manuscript, which demonstrate that IFN-β and -γ are dormancy drivers (190–193). To complicate the matter further, acute exposure of HSCs to IFN-I has been shown to induce quiescence exit and promote proliferation; however, far from leading to HSC pool exhaustion, chronic exposure to IFN-I reestablished the HSC quiescent state and induced protection from the killing effects of IFN-I (195). These findings highlight the importance of advancing research focused on IFN pathway regulation, since IFNs (specially IFN-α) have been proposed as “awakening” agents for dormant CSCs. Despite these findings described for HSC, it is yet to be demonstrated whether acute and chronic exposure of other CSCs to IFN-I induces the same effects as those described for HSCs, but caution should be taken when exploring the therapeutic effects of IFNs on CSCs, specifically at the level of dormancy.

**Figure 2:**
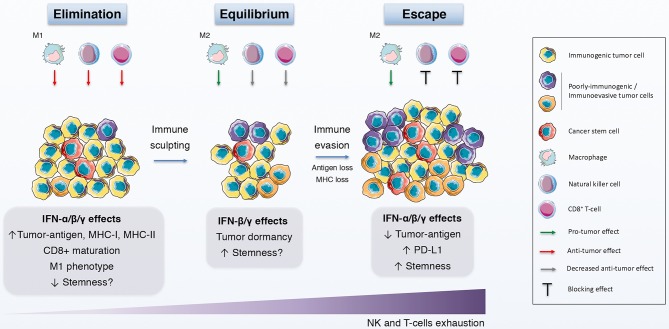
IFNs in the immunoediting process. Schematic representation of the immunoediting process, which is divided in the elimination, equilibrium and escape phases, and how IFNs affects this process. During the elimination phase, both innate and adaptive immune systems identify and eliminate immunogenic tumor cells with the help of the anti-tumor effects of IFN. The immune pressure gives rise to the selection of poorly-immunogenic tumor cells and to a static phase, the equilibrium, in which the growth and elimination of tumor cells is balanced and the quiescence state is promoted, in part by IFNs. Finally, tumor evolution favors the induction and selection of immunoevasive features on tumor cells, some of which are elicited by IFNs, thus driving tumor survival and growth.

Finally and more interestingly, the concept of immunoediting might prove beneficial to further explain the contradictory conclusions regarding the effect of IFNs on CSCs (196) (Figure 2) (Table 1). Briefly, cancer immunoediting refers to a complex interplay between tumor cells and the host immune system that can be divided into three phases: elimination (immunosurveillance), equilibrium (quiescent state) and escape (immunoevasion) [reviewed by McCoach and Bivona (197)]. Thus, depending on the molecular and functional traits of a CSC subset at a certain time during tumor progression, IFNs would be able to boost or shut down that subpopulation. Although knowledge of how CSCs participate in cancer immunoediting is now expanding (198, 199), less is known about the role of IFNs in that interplay. Exploring this field would surely contribute significantly to a better understanding of the dynamics and relationship that exists between IFNs and CSCs.

**Table 1:** Anti- and pro-CSC effects of IFN-I and IFN-II.

**Anti-CSC effects**	**Pro-CSC effects**
**IFN-I**	
Decreased expression of stem-like markers and/or pluripotency genes (1–3)	Increased expression of stem-like markers and/or pluripotency genes (4–6)
Reduced self-renewal capacity (1, 2, 7–10)	Increased self-renewal capacity (4–6)
Reduced tumorigenic potential (1, 7, 9)	Increased tumorigenic potential (5, 6)
Reduced proliferation (7, 9, 10)	Chemoresistance (6)
Reduced metastatic potential (9)	Increased migratory/invasive and/or metastatic capacities (3, 6)
	Induction of dormancy (11)
**IFN-II**	
Reduced self-renewal capacity (14)	Increased self-renewal capacity (12, 15)
Reduced tumorigenic potential (14)	Increased tumorigenic potential (13)
Reduced migratory/invasive capacities (16)	Activation of EMT and/or migration/invasion (12, 15)
	Increased expression of stem-like markers and/or pluripotency genes (12, 13)
	Increased metastatic potential (15, 17–22)
	Chemoresistance (15)
	Induction of dormancy (23–25)

In conclusion, IFNs comprise a family of cytokines with pleiotropic effects, and among the many effects attributed to IFNs and their signaling pathways, growing evidence now validates a unique role for these cytokines in CSC biology. IFNs are able to display both pro- and anti-CSCs effects, depending on the context, including synergistic effects with other cytokines. For this reason, further research is needed in order to build a more comprehensive perspective of these contradictory roles with the hope of being able to exploit the anti-tumor effects of IFNs and at the same time downregulate their pro-CSCs capabilities as a means of targeting CSCs to improve cancer patient overall survival.

## Author Contributions

BS and LM-H developed the idea and edited the text. LM-H wrote the manuscript. All authors listed have made a substantial, direct and intellectual contribution to the work, and approved it for publication.

### Conflict of Interest

The authors declare that the research was conducted in the absence of any commercial or financial relationships that could be construed as a potential conflict of interest.

## References

[1] Isaacs A Lindenmann J Andrewes CH Virus interference. I. The interferon. Proc R Soc Lond Ser B Biol Sci. (1957) 147:258–67. 10.1098/rspb.1957.0048 26297790

[2] de Weerd NA Nguyen T. The interferons and their receptors–distribution and regulation. Immunol Cell Biol. (2012) 90:483–91. 10.1038/icb.2012.9 22410872 PMC7165917

[3] Farrar MA Schreiber RD. The molecular cell biology of interferon-gamma and its Receptor. Annu Rev Immunol. (1993) 11:571–611. 10.1146/annurev.iy.11.040193.003035 8476573

[4] Kotenko SV Gallagher G Baurin VV Lewis-Antes A Shen M Shah NK. IFN-lambdas mediate antiviral protection through a distinct class II cytokine receptor complex. Nat Immunol. (2003) 4:69–77. 10.1038/ni875 12483210

[5] Majoros A Platanitis E Kernbauer-Hölzl E Rosebrock F Müller M Decker T. Canonical and non-canonical aspects of JAK–STAT signaling: lessons from interferons for cytokine responses. Front Immunol. (2017) 8:29. 10.3389/fimmu.2017.00029 28184222 PMC5266721

[6] Hooks JJ Jordan GW Cupps T Moutsopoulos HM Fauci AS Notkins AL. Multiple interferons in the circulation of patients with systemic lupus erythematosus and vasculitis. Arthritis Rheum. (1982) 25:396–400. 10.1002/art.1780250406 6176247

[7] Preble OT Black RJ Friedman RM Klippel JH Vilcek J. Systemic lupus erythematosus: presence in human serum of an unusual acid-labile leukocyte interferon. Science. (1982) 216:429–31. 10.1126/science.6176024 6176024

[8] Skurkovich SV Eremkina EI. The probable role of interferon in allergy. Ann Allergy. (1975) 35:356–60. 1200423

[9] Blanco P Palucka AK Gill M Pascual V Banchereau J. Induction of dendritic cell differentiation by IFN-alpha in systemic lupus erythematosus. Science. (2001) 294:1540–3. 10.1126/science.1064890 11711679

[10] van der Pouw Kraan TCTM Wijbrandts CA van Baarsen LGM Voskuyl AE Rustenburg F Baggen JM. Rheumatoid arthritis subtypes identified by genomic profiling of peripheral blood cells: assignment of a type I interferon signature in a subpopulation of patients. Ann Rheum Dis. (2007) 66:1008–14. 10.1136/ard.2006.063412 17223656 PMC1954704

[11] Castañeda-Delgado JE Bastián-Hernandez Y Macias-Segura N Santiago-Algarra D Castillo-Ortiz JD Alemán-Navarro AL. Type I interferon gene response is increased in early and established rheumatoid arthritis and correlates with autoantibody production. Front Immunol. (2017) 8:285. 10.3389/fimmu.2017.00285 28373872 PMC5357778

[12] Pestka S Krause CD Walter MR. Interferons, interferon-like cytokines, and their receptors. Immunol Rev. (2004) 202:8–32. 10.1111/j.0105-2896.2004.00204.x 15546383

[13] Uddin S Majchrzak B Woodson J Arunkumar P Alsayed Y Pine R. Activation of the p38 mitogen-activated protein kinase by type I interferons. J Biol Chem. (1999) 274:30127–31. 10.1074/jbc.274.42.30127 10514501

[14] David M Petricoin E Benjamin C Pine R Weber MJ Larner AC. Requirement for MAP kinase (ERK2) activity in interferon alpha- and interferon beta-stimulated gene expression through STAT proteins. Science. (1995) 269:1721–3. 10.1126/science.7569900 7569900

[15] Uddin S Fish EN Sher DA Gardziola C White MF Platanias LC. Activation of the phosphatidylinositol 3-kinase serine kinase by IFN-alpha. J Immunol. (1997) 158:2390–7. 9036989

[16] Uddin S Yenush L Sun XJ Sweet ME White MF Platanias LC. Interferon-alpha engages the insulin receptor substrate-1 to associate with the phosphatidylinositol 3'-kinase. J Biol Chem. (1995) 270:15938–41. 10.1074/jbc.270.27.15938 7608146

[17] Kaur S Uddin S Platanias LC. The PI3' kinase pathway in interferon signaling. J Interferon Cytokine Res. (2005) 25:780–7. 10.1089/jir.2005.25.780 16375606

[18] Yang CH Murti A Pfeffer SR Kim JG Donner DB Pfeffer LM Interferon alpha /beta promotes cell survival by activating nuclear factor kappa B through phosphatidylinositol 3-kinase and Akt. J Biol Chem. (2001) 276:13756–61. 10.1074/jbc.M011006200 11278812

[19] Du Z Wei L Murti A Pfeffer SR Fan M Yang CH. Non-conventional signal transduction by type 1 interferons: the NF-kappaB pathway. J Cell Biochem. (2007) 102:1087–94. 10.1002/jcb.21535 17910035

[20] Cheon H Stark GR. Unphosphorylated STAT1 prolongs the expression of interferon-induced immune regulatory genes. Proc Natl Acad Sci USA. (2009) 106:9373–8. 10.1073/pnas.0903487106 19478064 PMC2688000

[21] Schneider WM Chevillotte MD Rice CM. Interferon-Stimulated genes: a complex web of host defenses. Annu Rev Immunol. (2014) 32:513–45. 10.1146/annurev-immunol-032713-120231 24555472 PMC4313732

[22] Madera S Rapp M Firth MA Beilke JN Lanier LL Sun JC. Type I IFN promotes NK cell expansion during viral infection by protecting NK cells against fratricide. J Exp Med. (2016) 213:225–33. 10.1084/jem.20150712 26755706 PMC4749923

[23] Biron CA Sonnenfeld G Welsh RM. Interferon induces natural killer cell blastogenesis *in vivo*. J Leukoc Biol. (1984) 35:31–7. 10.1002/jlb.35.1.31 6200554

[24] Martinez J Huang X Yang Y. Direct action of type I IFN on NK cells is required for their activation in response to vaccinia viral infection *in vivo*. J Immunol. (2008) 180:1592–7. 10.4049/jimmunol.180.3.1592 18209055

[25] Le Bon A Etchart N Rossmann C Ashton M Hou S Gewert D. Cross-priming of CD8+ T cells stimulated by virus-induced type I interferon. Nat Immunol. (2003) 4:1009–15. 10.1038/ni978 14502286

[26] Brinkmann V Geiger T Alkan S Heusser CH. Interferon alpha increases the frequency of interferon gamma-producing human CD4+ T cells. J Exp Med. (1993) 178:1655–63. 10.1084/jem.178.5.1655 8228812 PMC2191249

[27] Marrack P Kappler J Mitchell T. Type I interferons keep activated t cells alive. J Exp Med. (1999) 189:521–30. 10.1084/jem.189.3.521 9927514 PMC2192920

[28] Kolumam GA Thomas S Thompson LJ Sprent J Murali-Krishna K. Type I interferons act directly on CD8 T cells to allow clonal expansion and memory formation in response to viral infection. J Exp Med. (2005) 202:637–50. 10.1084/jem.20050821 16129706 PMC2212878

[29] Curtsinger JM Valenzuela JO Agarwal P Lins D Mescher MF. Type I IFNs provide a third signal to CD8 T cells to stimulate clonal expansion and differentiation. J Immunol. (2005) 174:4465–9. 10.4049/jimmunol.174.8.4465 15814665

[30] Gessani S Conti L Cornò MD Belardelli F. Type I Interferons as regulators of human antigen presenting cell functions. Toxins. (2014) 6:1696–723. 10.3390/toxins6061696 24866026 PMC4073125

[31] Platanias LC. Mechanisms of type-I- and type-II-interferon-mediated signalling. Nat Rev Immunol. (2005) 5:375. 10.1038/nri1604 15864272

[32] Nguyen H Ramana CV Bayes J Stark GR. Roles of phosphatidylinositol 3-kinase in interferon-gamma-dependent phosphorylation of STAT1 on serine 727 and activation of gene expression. J Biol Chem. (2001) 276:33361–8. 10.1074/jbc.M105070200 11438544

[33] Rosner D Stoneman V Littlewood T McCarthy N Figg N Wang Y. Interferon-γ induces fas trafficking and sensitization to apoptosis in vascular smooth muscle cells via a PI3K- and Akt-dependent mechanism. Am J Pathol. (2006) 168:2054–63. 10.2353/ajpath.2006.050473 16723718 PMC1606618

[34] Lin Y Jamison S Lin W. Interferon-γ activates nuclear factor-κ B in oligodendrocytes through a process mediated by the unfolded protein response. PLoS ONE. (2012) 7:e36408. 10.1371/journal.pone.0036408 22574154 PMC3344863

[35] Gough DJ Levy DE Johnstone RW Clarke CJ. IFNgamma signaling-does it mean JAK-STAT? Cytokine Growth Factor Rev. (2008) 19:383–94. 10.1016/j.cytogfr.2008.08.004 18929502

[36] Dallagi A Girouard J Hamelin-Morrissette J Dadzie R Laurent L Vaillancourt C. The activating effect of IFN-γ on monocytes/macrophages is regulated by the LIF–trophoblast–IL-10 axis via Stat1 inhibition and Stat3 activation. Cell Mol Immunol. (2015) 12:326–41. 10.1038/cmi.2014.50 25027966 PMC4654315

[37] Wu C Xue Y Wang P Lin L Liu Q Li N. IFN-γ primes macrophage activation by increasing phosphatase and tensin homolog via downregulation of miR-3473b. J Immunol. (2014) 193:3036–44. 10.4049/jimmunol.1302379 25092892

[38] Herbst S Schaible UE Schneider BE. Interferon gamma activated macrophages kill mycobacteria by nitric oxide induced apoptosis. PLoS ONE. (2011) 6:e19105. 10.1371/journal.pone.0019105 21559306 PMC3085516

[39] Held TK Weihua X Yuan L Kalvakolanu DV Cross AS. Gamma interferon augments macrophage activation by lipopolysaccharide by two distinct mechanisms, at the signal transduction level and via an autocrine mechanism involving tumor necrosis factor alpha and interleukin-1. Infect Immun. (1999) 67:206–12. 10.1128/IAI.67.1.206-212.1999 9864217 PMC96298

[40] Haring JS Corbin GA Harty JT. Dynamic regulation of IFN-γ signaling in antigen-specific CD8+ T cells responding to infection. J Immunol. (2005) 174:6791–802. 10.4049/jimmunol.174.11.6791 15905520

[41] Refaeli Y Van Parijs L Alexander SI Abbas AK. Interferon γ is required for activation-induced death of T lymphocytes. J Exp Med. (2002) 196:999–1005. 10.1084/jem.20020666 12370261 PMC2194022

[42] Whitmire JK Tan JT Whitton JL. Interferon-γ acts directly on CD8+ T cells to increase their abundance during virus infection. J Exp Med. (2005) 201:1053–9. 10.1084/jem.20041463 15809350 PMC2213135

[43] Nathan CF Murray HW Wiebe ME Rubin BY. Identification of interferon-gamma as the lymphokine that activates human macrophage oxidative metabolism and antimicrobial activity. J Exp Med. (1983) 158:670–89. 10.1084/jem.158.3.670 6411853 PMC2187114

[44] MacMicking JD Taylor GA McKinney JD. Immune control of tuberculosis by IFN-gamma-inducible LRG-47. Science. (2003) 302:654–9. 10.1126/science.1088063 14576437

[45] Karupiah G Xie QW Buller RM Nathan C Duarte C MacMicking JD. Inhibition of viral replication by interferon-gamma-induced nitric oxide synthase. Science. (1993) 261:1445–8. 10.1126/science.7690156 7690156

[46] Collazo CM Yap GS Sempowski GD Lusby KC Tessarollo L Woude GFV. Inactivation of Lrg-47 and Irg-47 reveals a family of interferon γ-inducible genes with essential, pathogen-specific roles in resistance to infection. J Exp Med. (2001) 194:181–8. 10.1084/jem.194.2.181 11457893 PMC2193451

[47] Gao PQ Sims SH Chang DC Deisseroth AB. Interferon-gamma priming effects in the activation and deactivation of ISGF3 in K562 cells. J Biol Chem. (1993) 268:12380–7. 8509377

[48] Yoshida A Koide Y Uchijima M Yoshida TO. IFN-gamma induces IL-12 mRNA expression by a murine macrophage cell line, J774. Biochem Biophys Res Commun. (1994) 198:857–61. 10.1006/bbrc.1994.1122 7906942

[49] Gough DJ Messina NL Hii L Gould JA Sabapathy K Robertson APS. Functional crosstalk between type I and II Interferon through the regulated expression of STAT1. PLoS Biol. (2010) 8:e1000361. 10.1371/journal.pbio.1000361 20436908 PMC2860501

[50] Hu X Ivashkiv LB. Cross-regulation of signaling pathways by interferon-γ: implications for immune responses and autoimmune diseases. Immunity. (2009) 31:539–50. 10.1016/j.immuni.2009.09.002 19833085 PMC2774226

[51] Zhang Y Apilado R Coleman J Ben-Sasson S Tsang S Hu-Li J. Interferon gamma stabilizes the T helper cell type 1 phenotype. J Exp Med. (2001) 194:165–72. 10.1084/jem.194.2.165 11457891 PMC2193457

[52] Tau GZ von der Weid T Lu B Cowan S Kvatyuk M Pernis A. Interferon γ signaling alters the function of T helper type 1 cells. J Exp Med. (2000) 192:977–86. 10.1084/jem.192.7.977 11015439 PMC2193318

[53] Wensky A Marcondes MCG Lafaille JJ. The role of IFN-γ in the production of Th2 subpopulations: implications for variable Th2-mediated pathologies in autoimmunity. J Immunol. (2001) 167:3074–81. 10.4049/jimmunol.167.6.3074 11544291

[54] Mohr E Cunningham AF Toellner K-M Bobat S Coughlan RE Bird RA. IFN-γ produced by CD8 T cells induces T-bet–dependent and –independent class switching in B cells in responses to alum-precipitated protein vaccine. Proc Natl Acad Sci USA. (2010) 107:17292–7. 10.1073/pnas.1004879107 20855629 PMC2951392

[55] Bossie A Vitetta ES. IFN-gamma enhances secretion of IgG2a from IgG2a-committed LPS-stimulated murine B cells: implications for the role of IFN-gamma in class switching. Cell Immunol. (1991) 135:95–104. 10.1016/0008-8749(91)90257-C 1902147

[56] Melero I Rouzaut A Motz GT Coukos G. T-cell and NK-cell infiltration into solid tumors: a key limiting factor for efficacious cancer immunotherapy. Cancer Discov. (2014) 4:522–6. 10.1158/2159-8290.CD-13-0985 24795012 PMC4142435

[57] Pak-Wittel MA Yang L Sojka DK Rivenbark JG Yokoyama WM. Interferon-γ mediates chemokine-dependent recruitment of natural killer cells during viral infection. Proc Natl Acad Sci USA. (2013) 110:E50–9. 10.1073/pnas.1220456110 23248310 PMC3538256

[58] Aquino-López A Senyukov VV Vlasic Z Kleinerman ES Lee DA. Interferon gamma induces changes in Natural Killer (NK) cell ligand expression and alters NK Cell-mediated lysis of pediatric cancer cell lines. Front Immunol. (2017) 8:391. 10.3389/fimmu.2017.00391 28428785 PMC5382194

[59] Chawla-Sarkar M Leaman DW Borden EC. Preferential induction of apoptosis by interferon (IFN)-beta compared with IFN-alpha2: correlation with TRAIL/Apo2L induction in melanoma cell lines. Clin Cancer Res. (2001) 7:1821–31. Available online at: https://clincancerres.aacrjournals.org/11410525

[60] Cheriyath V Glaser KB Waring JF Baz R Hussein MA Borden EC. G1P3, an IFN-induced survival factor, antagonizes TRAIL-induced apoptosis in human myeloma cells. J Clin Invest. (2007) 117:3107–17. 10.1172/JCI31122 17823654 PMC1964509

[61] Bernassola F Scheuerpflug C Herr I Krammer PH Debatin K-M Melino G. Induction of apoptosis by IFNγ in human neuroblastoma cell lines through the CD95/CD95L autocrine circuit. Cell Death Differ. (1999) 6:652–60. 10.1038/sj.cdd.4400537 10453076

[62] Ugurel S Seiter S Rappl G Stark A Tilgen W Reinhold U. Heterogenous susceptibility to CD95-induced apoptosis in melanoma cells correlates with bcl-2 and bcl-x expression and is sensitive to modulation by interferon-γ. Int J Cancer. (1999) 82:727–36. 10.1002/(SICI)1097-0215(19990827)82:5<727::AID-IJC17>3.0.CO;2-E 10417772

[63] Kotredes KP Gamero AM. Interferons as inducers of apoptosis in malignant cells. J Interferon Cytokine Res. (2013) 33:162–70. 10.1089/jir.2012.0110 23570382 PMC3624694

[64] Shang D Yang P Liu Y Song J Zhang F Tian Y. Interferon-α induces G1 cell-cycle arrest in renal cell carcinoma cells via activation of Jak-Stat signaling. Cancer Invest. (2011) 29:347–52. 10.3109/07357907.2011.568566 21599510

[65] Bekisz J Baron S Balinsky C Morrow A Zoon KC. Antiproliferative properties of type I and type II interferon. Pharmaceuticals. (2010) 3:994–1015. 10.3390/ph3040994 20664817 PMC2907165

[66] Rosewicz S Detjen K Scholz A von Marschall Z. Interferon-alpha: regulatory effects on cell cycle and angiogenesis. Neuroendocrinology. (2004) 80(Suppl. 1):85–93. 10.1159/000080748 15477724

[67] Sangfelt O Erickson S Grander D. Mechanisms of interferon-induced cell cycle arrest. Front Biosci. (2000) 5:D479–87. 10.2741/A527 10762599

[68] von Marschall Z Scholz A Cramer T Schäfer G Schirner M Oberg K. Effects of interferon alpha on vascular endothelial growth factor gene transcription and tumor angiogenesis. J Natl Cancer Inst. (2003) 95:437–48. 10.1093/jnci/95.6.437 12644537

[69] Sato N Nariuchi H Tsuruoka N Nishihara T Beitz JG Calabresi P. Actions of TNF and IFN-gamma on angiogenesis *in vitro*. J Invest Dermatol. (1990) 95:85S−9S. 10.1111/1523-1747.ep12874809 1701814

[70] Indraccolo S. Interferon-alpha as angiogenesis inhibitor: learning from tumor models. Autoimmunity. (2010) 43:244–7. 10.3109/08916930903510963 20166871

[71] Takano S Ishikawa E Matsuda M Yamamoto T Matsumura A. Interferon-β inhibits glioma angiogenesis through downregulation of vascular endothelial growth factor and upregulation of interferon inducible protein 10. Int J Oncol. (2014) 45:1837–46. 10.3892/ijo.2014.2620 25175315 PMC4203325

[72] Sun T Yang Y Luo X Cheng Y Zhang M Wang K. Inhibition of tumor angiogenesis by interferon-γ by suppression of tumor-associated macrophage differentiation. Oncol Res. (2014) 21:227–35. 10.3727/096504014X13890370410285 24854099

[73] Friesel R Komoriya A Maciag T. Inhibition of endothelial cell proliferation by gamma-interferon. J Cell Biol. (1987) 104:689–96. 10.1083/jcb.104.3.689 3102503 PMC2114528

[74] Albini A Marchisone C Del Grosso F Benelli R Masiello L Tacchetti C. Inhibition of angiogenesis and vascular tumor growth by interferon-Producing cells. Am J Pathol. (2000) 156:1381–93. 10.1016/S0002-9440(10)65007-9 10751362 PMC1876903

[75] Boyer CM Dawson DV Neal SE Winchell LF Leslie DS Ring D. Differential induction by interferons of major histocompatibility complex-encoded and non-major histocompatibility complex-encoded antigens in human breast and ovarian carcinoma cell lines. Cancer Res. (1989) 49:2928–34. 2497969

[76] Greiner JW Hand PH Noguchi P Fisher PB Pestka S Schlom J. Enhanced expression of surface tumor-associated antigens on human breast and colon tumor cells after recombinant human leukocyte alpha-interferon treatment. Cancer Res. (1984) 44:3208–14. 10.1016/B978-0-08-031739-7.50204-4 6744259

[77] Dolei A Capobianchi MR Ameglio F. Human interferon-gamma enhances the expression of class I and class II major histocompatibility complex products in neoplastic cells more effectively than interferon-alpha and interferon-beta. Infect Immun. (1983) 40:172–6. 10.1128/IAI.40.1.172-176.1983 6299957 PMC264832

[78] Schiavoni G Mattei F Gabriele L. Type I interferons as stimulators of DC-mediated cross-priming: impact on anti-tumor response. Front Immunol. (2013) 4:483. 10.3389/fimmu.2013.00483 24400008 PMC3872318

[79] Curtsinger JM Mescher MF. Inflammatory cytokines as a third signal for T cell activation. Curr Opin Immunol. (2010) 22:333–40. 10.1016/j.coi.2010.02.013 20363604 PMC2891062

[80] Fuertes MB Kacha AK Kline J Woo S-R Kranz DM Murphy KM. Host type I IFN signals are required for antitumor CD8+ T cell responses through CD8{alpha}+ dendritic cells. J Exp Med. (2011) 208:2005–16. 10.1084/jem.20101159 21930765 PMC3182064

[81] Pace L Vitale S Dettori B Palombi C La Sorsa V Belardelli F. APC activation by IFN-alpha decreases regulatory T cell and enhances Th cell functions. J Immunol. (2010) 184:5969–79. 10.4049/jimmunol.0900526 20427775

[82] Srivastava S Koch MA Pepper M Campbell DJ. Type I interferons directly inhibit regulatory T cells to allow optimal antiviral T cell responses during acute LCMV infection. J Exp Med. (2014) 211:961–74. 10.1084/jem.20131556 24711580 PMC4010906

[83] Duluc D Corvaisier M Blanchard S Catala L Descamps P Gamelin E. Interferon-gamma reverses the immunosuppressive and protumoral properties and prevents the generation of human tumor-associated macrophages. Int J Cancer. (2009) 125:367–73. 10.1002/ijc.24401 19378341

[84] Watanabe MAE Oda JMM Amarante MK Cesar Voltarelli J. Regulatory T cells and breast cancer: implications for immunopathogenesis. Cancer Metastasis Rev. (2010) 29:569–79. 10.1007/s10555-010-9247-y 20830504

[85] Parker BS Rautela J Hertzog PJ. Antitumour actions of interferons: implications for cancer therapy. Nat Rev Cancer. (2016) 16:131–44. 10.1038/nrc.2016.14 26911188

[86] Yang CH Murti A Pfeffer SR Basu L Kim JG Pfeffer LM IFNα/β promotes cell survival by activating NF-κB. Proc Natl Acad Sci USA. (2000) 97:13631–6. 10.1073/pnas.250477397 11095741 PMC17627

[87] Pfeffer LM. The role of nuclear factor κB in the interferon response. J Interferon Cytokine Res. (2011) 31:553–9. 10.1089/jir.2011.0028 21631354 PMC3128784

[88] Puthier D Thabard W Rapp M Etrillard M Harousseau J Bataille R. Interferon alpha extends the survival of human myeloma cells through an upregulation of the Mcl-1 anti-apoptotic molecule. Br J Haematol. (2001) 112:358–63. 10.1046/j.1365-2141.2001.02575.x 11167829

[89] Cheriyath V Kuhns MA Jacobs BS Evangelista P Elson P Downs-Kelly E. G1P3, an interferon- and estrogen-induced survival protein contributes to hyperplasia, tamoxifen resistance and poor outcomes in breast cancer. Oncogene. (2012) 31:2222–36. 10.1038/onc.2011.393 21996729

[90] Asao H Fu X-Y Interferon-γ has dual potentials in inhibiting or promoting cell proliferation. J Biol Chem. (2000) 275:867–74. 10.1074/jbc.275.2.867 10625620

[91] Yan J Jiang Y Lu J Wu J Zhang M. Inhibiting of proliferation, migration, and invasion in lung cancer induced by silencing interferon-induced transmembrane protein 1 (IFITM1). BioMed Res Int. (2019) 2019:9085435. 10.1155/2019/9085435 31205947 PMC6530206

[92] Hatano H Kudo Y Ogawa I Tsunematsu T Kikuchi A Abiko Y. IFN-induced transmembrane protein 1 promotes invasion at early stage of head and neck cancer progression. Clin Cancer Res. (2008) 14:6097–105. 10.1158/1078-0432.CCR-07-4761 18829488

[93] Gomez D Reich NC. Stimulation of primary human endothelial cell proliferation by IFN. J Immunol. (2003) 170:5373–81. 10.4049/jimmunol.170.11.5373 12759411

[94] Duarte CW Willey CD Zhi D Cui X Harris JJ Vaughan LK. Expression signature of IFN/STAT1 signaling genes predicts poor survival outcome in glioblastoma multiforme in a subtype-specific manner. PLoS ONE. (2012) 7:e29653. 10.1371/journal.pone.0029653 22242177 PMC3252343

[95] Weichselbaum RR Ishwaran H Yoon T Nuyten DSA Baker SW Khodarev N. An interferon-related gene signature for DNA damage resistance is a predictive marker for chemotherapy and radiation for breast cancer. Proc Natl Acad Sci USA. (2008) 105:18490–5. 10.1073/pnas.0809242105 19001271 PMC2587578

[96] Khodarev NN Roach P Pitroda SP Golden DW Bhayani M Shao MY. STAT1 pathway mediates amplification of metastatic potential and resistance to therapy. PLoS ONE. (2009) 4:e5821. 10.1371/journal.pone.0005821 19503789 PMC2688034

[97] Taniguchi K Petersson M Höglund P Kiessling R Klein G Kärre K. Interferon gamma induces lung colonization by intravenously inoculated B16 melanoma cells in parallel with enhanced expression of class I major histocompatibility complex antigens. Proc Natl Acad Sci USA. (1987) 84:3405–9. 10.1073/pnas.84.10.3405 3106968 PMC304879

[98] Beatty GL Paterson Y. IFN-γ can promote tumor evasion of the immune system *in vivo* by down-regulating cellular levels of an endogenous tumor antigen. J Immunol. (2000) 165:5502–8. 10.4049/jimmunol.165.10.5502 11067903

[99] Le Poole IC Riker AI Quevedo ME Stennett LS Wang E Marincola FM. Interferon-gamma reduces melanosomal antigen expression and recognition of melanoma cells by cytotoxic T cells. Am J Pathol. (2002) 160:521–8. 10.1016/S0002-9440(10)64871-7 11839572 PMC1850638

[100] Abiko K Matsumura N Hamanishi J Horikawa N Murakami R Yamaguchi K. IFN-γ from lymphocytes induces PD-L1 expression and promotes progression of ovarian cancer. Br J Cancer. (2015) 112:1501–9. 10.1038/bjc.2015.101 25867264 PMC4453666

[101] Karasar P Esendagli G. T helper responses are maintained by basal-like breast cancer cells and confer to immune modulation via upregulation of PD-1 ligands. Breast Cancer Res Treat. (2014) 145:605–14. 10.1007/s10549-014-2984-9 24816762

[102] Provance OK Lewis-Wambi J. Deciphering the role of interferon alpha signaling and microenvironment crosstalk in inflammatory breast cancer. Breast Cancer Res. (2019) 21:59. 10.1186/s13058-019-1140-1 31060575 PMC6501286

[103] FDA-Approved Cancer Immunotherapies and CRI's Impact Cancer Res Inst. Available online at: https://www.cancerresearch.org/blog/april-2015/fda-approved-cancer-immunotherapies-cris-impact (accessed October 10, 2019).

[104] Talpaz M Hehlmann R Quintás-Cardama A Mercer J Cortes J. Re-emergence of interferon-α in the treatment of chronic myeloid leukemia. Leukemia. (2013) 27:803–12. 10.1038/leu.2012.313 23238589 PMC3703612

[105] Vacchelli E Aranda F Obrist F Eggermont A Galon J Cremer I. Trial watch: immunostimulatory cytokines in cancer therapy. Oncoimmunology. (2014) 3:e29030. 10.4161/onci.29030 25083328 PMC4091551

[106] Zitvogel L Galluzzi L Kepp O Smyth MJ Kroemer G. Type I interferons in anticancer immunity. Nat Rev Immunol. (2015) 15:405–14. 10.1038/nri3845 26027717

[107] Namikawa K Tsutsumida A Mizutani T Shibata T Takenouchi T Yoshikawa S. Randomized phase III trial of adjuvant therapy with locoregional interferon beta versus surgery alone in stage II/III cutaneous melanoma: Japan clinical oncology group study (JCOG1309, J-FERON). Jpn J Clin Oncol. (2017) 47:664–7. 10.1093/jjco/hyx063 29136453 PMC5896686

[108] Nakamura Y Tanaka K Shibata T Mizusawa J Mizutani T Fukuda H Confirmatory trial of non-amputative digit preservation surgery in subungual melanoma: JCOG1602 (J-NAIL study). J Clin Oncol. (2018) 36:TPS9607 10.1200/JCO.2018.36.15_suppl.TPS9607 PMC6815042 31653251

[109] Kawaji H Tokuyama T Yamasaki T Amano S Sakai N Namba H. Interferon-β and temozolomide combination therapy for temozolomide monotherapy-refractory malignant gliomas. Mol Clin Oncol. (2015) 3:909–13. 10.3892/mco.2015.542 26171205 PMC4486788

[110] Wakabayashi T Natsume A Mizusawa J Katayama H Fukuda H Sumi M. JCOG0911 INTEGRA study: a randomized screening phase II trial of interferonβ plus temozolomide in comparison with temozolomide alone for newly diagnosed glioblastoma. J Neurooncol. (2018) 138:627–36. 10.1007/s11060-018-2831-7 29557060 PMC5999164

[111] Repetto L Giannessi PG Campora E Pronzato P Vigani A Naso C. Tamoxifen and interferon-beta for the treatment of metastatic breast cancer. Breast Cancer Res Treat. (1996) 39:235–8. 10.1007/BF01806190 8872332

[112] Windbichler GH Hausmaninger H Stummvoll W Graf AH Kainz C Lahodny J. Interferon-gamma in the first-line therapy of ovarian cancer: a randomized phase III trial. Br J Cancer. (2000) 82:1138–44. 10.1054/bjoc.1999.1053 10735496 PMC2363351

[113] Giannopoulos A Constantinides C Fokaeas E Stravodimos C Giannopoulou M Kyroudi A. The immunomodulating effect of interferon-gamma intravesical instillations in preventing bladder cancer recurrence. Clin Cancer Res. (2003) 9:5550–8. Available online at: https://clincancerres.aacrjournals.org/14654535

[114] Creagan ET Schaid DJ Ahmann DL Frytak S. Disseminated malignant melanoma and recombinant interferon: analysis of seven consecutive phase II investigations. J Invest Dermatol. (1990) 95:188S−92S. 10.1111/1523-1747.ep12875512 2124246

[115] Talpaz M Kurzrock R Kantarjian H Rothberg J Saks S Evans L. A phase II study alternating alpha-2a-interferon and gamma-interferon therapy in patients with chronic myelogenous leukemia. Cancer. (1991) 68:2125–30. 10.1002/1097-0142(19911115)68:10&lt 1913450

[116] Brown TD Goodman PJ Fleming T Macdonald JS O'Rourke T Taylor SA. Phase II trial of recombinant DNA gamma-interferon in advanced colorectal cancer: a southwest oncology group study. J Immunother. (1991) 10:379–82. 10.1097/00002371-199110000-00011 1790147

[117] Von Hoff DD Fleming TR Macdonald JS Goodman PJ Van Damme J Brown TD. Phase II evaluation of recombinant gamma-interferon in patients with advanced pancreatic carcinoma: a southwest oncology group study. J Biol Response Mod. (1990) 9:584–7. 2127424

[118] Lollini PL Bosco MC Cavallo F De Giovanni C Giovarelli M Landuzzi L. Inhibition of tumor growth and enhancement of metastasis after transfection of the gamma-interferon gene. Int J Cancer. (1993) 55:320–9. 10.1002/ijc.2910550224 8370628

[119] Aul C Gattermann N Germing U Heyll A Adverse Effects of Interferon Treatment. In: Aul C, Schneider W, editors. Interferons. Heidelberg: Springer (1997). p. 250–66. 10.1007/978-3-642-60411-9_15

[120] Pinto EF Andrade C. Interferon-related depression: a primer on mechanisms, treatment, and prevention of a common clinical problem. Curr Neuropharmacol. (2016) 14:743–8. 10.2174/1570159X14666160106155129 26733280 PMC5050402

[121] Valle S Martin-Hijano L Alcalá S Alonso-Nocelo M Sainz B. Jr. The ever-evolving concept of the cancer stem cell in pancreatic cancer. Cancers. (2018) 10:33. 10.3390/cancers10020033 29373514 PMC5836065

[122] Rich JN. Cancer stem cells: understanding tumor hierarchy and heterogeneity. Medicine (Baltimore). (2016) 95 (1 Suppl. 1):S2–7. 10.1097/MD.0000000000004764 27611934 PMC5599210

[123] Nguyen LV Vanner R Dirks P Eaves CJ. Cancer stem cells: an evolving concept. Nat Rev Cancer. (2012) 12:133–43. 10.1038/nrc3184 22237392

[124] Ding B Liu P Liu W Sun P Wang C-L. Emerging roles of krüppel-like factor 4 in cancer and cancer stem cells. Asian Pac J Cancer Prev. (2015) 16:3629–33. 10.7314/APJCP.2015.16.9.3629 25987013

[125] Wang M-L Chiou S-H Wu C-W. Targeting cancer stem cells: emerging role of nanog transcription factor. OncoTargets Ther. (2013) 6:1207–20. 10.2147/OTT.S38114 24043946 PMC3772775

[126] Rizzino A Sox2 and Oct-3/4: a versatile pair of master regulators that orchestrate the self-renewal and pluripotency of embryonic stem cells by functioning as molecular rheostats. Wiley Interdiscip Rev Syst Biol Med. (2009) 1:228–36. 10.1002/wsbm.12 20016762 PMC2794141

[127] Takeda K Mizushima T Yokoyama Y Hirose H Wu X Qian Y. Sox2 is associated with cancer stem-like properties in colorectal cancer. Sci Rep. (2018) 8:17639. 10.1038/s41598-018-36251-0 30518951 PMC6281572

[128] Lonardo E Hermann PC Mueller M-T Huber S Balic A Miranda-Lorenzo I. Nodal/Activin signaling drives self-renewal and tumorigenicity of pancreatic cancer stem cells and provides a target for combined drug therapy. Cell Stem Cell. (2011) 9:433–46. 10.1016/j.stem.2011.10.001 22056140

[129] Karsten U Goletz S. What makes cancer stem cell markers different? Springerplus. (2013) 2:301. 10.1186/2193-1801-2-301 23888272 PMC3710573

[130] Li W Ma H Zhang J Zhu L Wang C Yang Y. Unraveling the roles of CD44/CD24 and ALDH1 as cancer stem cell markers in tumorigenesis and metastasis. Sci Rep. (2017) 7:13856. 10.1038/s41598-017-14364-2 29062075 PMC5653849

[131] Clarke MF Dick JE Dirks PB Eaves CJ Jamieson CHM Jones DL. Cancer stem cells–perspectives on current status and future directions: AACR workshop on cancer stem cells. Cancer Res. (2006) 66:9339–44. 10.1158/0008-5472.CAN-06-3126 16990346

[132] Prager BC Xie Q Bao S Rich JN. Cancer stem cells: the architects of the tumor ecosystem. Cell Stem Cell. (2019) 24:41–53. 10.1016/j.stem.2018.12.009 30609398 PMC6350931

[133] Grosse-Wilde A Fouquier d'Hérouël A McIntosh E Ertaylan G Skupin A Kuestner RE. Stemness of the hybrid epithelial/mesenchymal state in breast cancer and its association with poor survival. PLoS ONE. (2015) 10:e0126522. 10.1371/journal.pone.0126522 26020648 PMC4447403

[134] Jolly MK Boareto M Huang B Jia D Lu M Ben-Jacob E. Implications of the hybrid epithelial/mesenchymal phenotype in metastasis. Front Oncol. (2015) 5:155. 10.3389/fonc.2015.00155 26258068 PMC4507461

[135] Jolly MK Somarelli JA Sheth M Biddle A Tripathi SC Armstrong AJ. Hybrid epithelial/mesenchymal phenotypes promote metastasis and therapy resistance across carcinomas. Pharmacol Ther. (2019) 194:161–84. 10.1016/j.pharmthera.2018.09.007 30268772

[136] Chen W Dong J Haiech J Kilhoffer M-C Zeniou M. Cancer stem cell quiescence and plasticity as major challenges in cancer therapy. Stem Cells Int. (2016) 2016:1740936. 10.1155/2016/1740936 27418931 PMC4932171

[137] Dembinski JL Krauss S. Characterization and functional analysis of a slow cycling stem cell-like subpopulation in pancreas adenocarcinoma. Clin Exp Metastasis. (2009) 26:611–23. 10.1007/s10585-009-9260-0 19421880 PMC2776152

[138] Zhao J. Cancer stem cells and chemoresistance: the smartest survives the raid. Pharmacol Ther. (2016) 160:145–58. 10.1016/j.pharmthera.2016.02.008 26899500 PMC4808328

[139] Izumiya M Kabashima A Higuchi H Igarashi T Sakai G Iizuka H. Chemoresistance is associated with cancer stem cell-like properties and epithelial-to-mesenchymal transition in pancreatic cancer cells. Anticancer Res. (2012) 32:3847–53. Available online at: http://ar.iiarjournals.org/22993328

[140] Begicevic R-R Falasca M. ABC transporters in cancer stem cells: beyond chemoresistance. Int J Mol Sci. (2017) 18:2362. 10.3390/ijms18112362 29117122 PMC5713331

[141] Fletcher JI Haber M Henderson MJ Norris MD. ABC transporters in cancer: more than just drug efflux pumps. Nat Rev Cancer. (2010) 10:147–56. 10.1038/nrc2789 20075923

[142] Abdullah LN Chow EK-H. Mechanisms of chemoresistance in cancer stem cells. Clin Transl Med. (2013) 2:3. 10.1186/2001-1326-2-3 23369605 PMC3565873

[143] Castiello L Sestili P Schiavoni G Dattilo R Monque DM Ciaffoni F. Disruption of IFN-I signaling promotes HER2/Neu tumor progression and breast cancer stem cells. Cancer Immunol Res. (2018) 6:658–70. 10.1158/2326-6066.CIR-17-0675 29622580

[144] Doherty MR Cheon H Junk DJ Vinayak S Varadan V Telli ML. Interferon-beta represses cancer stem cell properties in triple-negative breast cancer. Proc Natl Acad Sci USA. (2017) 114:13792–7. 10.1073/pnas.1713728114 29229854 PMC5748193

[145] Yuki K Natsume A Yokoyama H Kondo Y Ohno M Kato T. Induction of oligodendrogenesis in glioblastoma-initiating cells by IFN-mediated activation of STAT3 signaling. Cancer Lett. (2009) 284:71–9. 10.1016/j.canlet.2009.04.020 19457609

[146] Bonni A Sun Y Nadal-Vicens M Bhatt A Frank DA Rozovsky I. Regulation of gliogenesis in the central nervous system by the JAK-STAT signaling pathway. Science. (1997) 278:477–83. 10.1126/science.278.5337.477 9334309

[147] Rajan P McKay RDG. Multiple routes to astrocytic differentiation in the CNS. J Neurosci. (1998) 18:3620–9. 10.1523/JNEUROSCI.18-10-03620.1998 9570793 PMC6793143

[148] Cimini A Cerù MP. Emerging roles of peroxisome proliferator-activated receptors (PPARs) in the regulation of neural stem cells proliferation and differentiation. Stem Cell Rev. (2008) 4:293–303. 10.1007/s12015-008-9024-2 18561036

[149] Hackett AR Lee D-H Dawood A Rodriguez M Funk L Tsoulfas P. STAT3 and SOCS3 regulate NG2 cell proliferation and differentiation after contusive spinal cord injury. Neurobiol Dis. (2016) 89:10–22. 10.1016/j.nbd.2016.01.017 26804026 PMC4785033

[150] Spiotto MT Chung TD. STAT3 mediates IL-6-induced neuroendocrine differentiation in prostate cancer cells. Prostate. (2000) 42:186–95. 10.1002/(SICI)1097-0045(20000215)42:3&lt 10639189

[151] Marotta LLC Almendro V Marusyk A Shipitsin M Schemme J Walker SR The JAK2/STAT3 signaling pathway is required for growth of CD44^+^CD24^−^ stem cell-like breast cancer cells in human tumors. J Clin Invest. (2011) 121:2723–35. 10.1172/JCI44745 21633165 PMC3223826

[152] Chung SS Aroh C Vadgama JV. Constitutive activation of STAT3 signaling regulates hTERT and promotes stem cell-like traits in human breast cancer cells. PLoS ONE. (2013) 8:e83971. 10.1371/journal.pone.0083971 24386318 PMC3875492

[153] Lin L Fuchs J Li C Olson V Bekaii-Saab T Lin J. STAT3 signaling pathway is necessary for cell survival and tumorsphere forming capacity in ALDH^+^/CD133^+^ stem cell-like human colon cancer cells. Biochem Biophys Res Commun. (2011) 416:246–51. 10.1016/j.bbrc.2011.10.112 22074823

[154] Schroeder A Herrmann A Cherryholmes G Kowolik C Buettner R Pal S. Loss of androgen receptor expression promotes a stem-like cell phenotype in prostate cancer through STAT3 signaling. Cancer Res. (2014) 74:1227–37. 10.1158/0008-5472.CAN-13-0594 24177177 PMC4539262

[155] Hajimoradi M Hassan ZM Ebrahimi M Soleimani M Bakhshi M Firouzi J. STAT3 is overactivated in gastric cancer stem-like cells. Cell J Yakhteh. (2017) 17:617–28. 10.22074/cellj.2016.3834 26862521 PMC4746412

[156] Du Z Cai C Sims M Boop FA Davidoff AM Pfeffer LM. The effects of type I interferon on glioblastoma cancer stem cells. Biochem Biophys Res Commun. (2017) 491:343–8. 10.1016/j.bbrc.2017.07.098 28728846

[157] Lykhova AA Kudryavets YI Strokovska LI Bezdenezhnykh NA Semesiuk NI Adamenko IN. Suppression of proliferation, tumorigenicity and metastasis of lung cancer cells after their transduction by interferon-beta gene in baculovirus vector. Cytokine. (2015) 71:318–26. 10.1016/j.cyto.2014.10.029 25497739

[158] Shimoda M Ota M Okada Y. Isolation of cancer stem cells by side population method. Methods Mol Biol. (2018) 1692:49–59. 10.1007/978-1-4939-7401-6_5 28986886

[159] Ma H Jin S Yang W Tian Z Liu S Wang Y. Interferon-α promotes the expression of cancer stem cell markers in oral squamous cell carcinoma. J Cancer. (2017) 8:2384–93. 10.7150/jca.19486 28819442 PMC5560157

[160] Qadir AS Ceppi P Brockway S Law C Mu L Khodarev NN. CD95/Fas increases stemness in cancer cells by inducing a STAT1-dependent type I interferon response. Cell Rep. (2017) 18:2373–86. 10.1016/j.celrep.2017.02.037 28273453 PMC5474321

[161] Ceppi P Hadji A Kohlhapp FJ Pattanayak A Hau A Liu X. CD95 and CD95L promote and protect cancer stem cells. Nat Commun. (2014) 5:5238. 10.1038/ncomms6238 25366259 PMC4417339

[162] Sainz B Martín B Tatari M Heeschen C Guerra S. ISG15 is a critical microenvironmental factor for pancreatic cancer stem cells. Cancer Res. (2014) 74:7309–20. 10.1158/0008-5472.CAN-14-1354 25368022

[163] Zhang D Zhang D-E. Interferon-stimulated gene 15 and the protein ISGylation system. J Interferon Cytokine Res. (2011) 31:119–30. 10.1089/jir.2010.0110 21190487 PMC3021351

[164] Chen R-H Du Y Han P Wang H-B Liang F-Y Feng G-K. ISG15 predicts poor prognosis and promotes cancer stem cell phenotype in nasopharyngeal carcinoma. Oncotarget. (2016) 7:16910–22. 10.18632/oncotarget.7626 26919245 PMC4941359

[165] Tsai Y-C Pestka S Wang L-H Runnels LW Wan S Lyu YL. Interferon-β signaling contributes to Ras transformation. PLoS ONE. (2011) 6:e24291. 10.1371/journal.pone.0024291 21897875 PMC3163666

[166] Sistigu A Yamazaki T Vacchelli E Chaba K Enot DP Adam J. Cancer cell–autonomous contribution of type I interferon signaling to the efficacy of chemotherapy. Nat Med. (2014) 20:1301–9. 10.1038/nm.3708 25344738

[167] Ogony J Choi HJ Lui A Cristofanilli M Lewis-Wambi J. Interferon-induced transmembrane protein 1 (IFITM1) overexpression enhances the aggressive phenotype of SUM149 inflammatory breast cancer cells in a signal transducer and activator of transcription 2 (STAT2)-dependent manner. Breast Cancer Res BCR. (2016) 18:25. 10.1186/s13058-016-0683-7 26897526 PMC4761146

[168] Monsurrò V Beghelli S Wang R Barbi S Coin S Di Pasquale G. Anti-viral state segregates two molecular phenotypes of pancreatic adenocarcinoma: potential relevance for adenoviral gene therapy. J Transl Med. (2010) 8:10. 10.1186/1479-5876-8-10 20113473 PMC2845551

[169] Ni C Wu P Zhu X Ye J Zhang Z Chen Z. IFN-γ selectively exerts pro-apoptotic effects on tumor-initiating label-retaining colon cancer cells. Cancer Lett. (2013) 336:174–84. 10.1016/j.canlet.2013.04.029 23643941

[170] Song M Ping Y Zhang K Yang L Li F Zhang C Low-dose IFN-γ induces tumor cell stemness in the tumor microenvironment of non-small cell lung cancer. Cancer Res. (2019) 79:canres0596.2019 10.1158/0008-5472.CAN-19-0596 31085700

[171] Bi J Tian Z. NK cell exhaustion. Front Immunol. (2017) 8:760. 10.3389/fimmu.2017.00760 28702032 PMC5487399

[172] Wherry EJ. T cell exhaustion. Nat Immunol. (2011) 12:492–9. 10.1038/ni.2035 21739672

[173] Matteucci C Balestrieri E Argaw-Denboba A Sinibaldi-Vallebona P. Human endogenous retroviruses role in cancer cell stemness. Semin Cancer Biol. (2018) 53:17–30. 10.1016/j.semcancer.2018.10.001 30317035

[174] Cañadas I Thummalapalli R Kim JW Kitajima S Jenkins RW Christensen CL. Tumor innate immunity primed by specific interferon-stimulated endogenous retroviruses. Nat Med. (2018) 24:1143–50. 10.1038/s41591-018-0116-5 30038220 PMC6082722

[175] Li S Xie Y Zhang W Gao J Wang M Zheng G. Interferon alpha-inducible protein 27 promotes epithelial-mesenchymal transition and induces ovarian tumorigenicity and stemness. J Surg Res. (2015) 193:255–64. 10.1016/j.jss.2014.06.055 25103640

[176] Mani SA Guo W Liao M-J Eaton EN Ayyanan A Zhou AY. The epithelial-mesenchymal transition generates cells with properties of stem cells. Cell. (2008) 133:704–15. 10.1016/j.cell.2008.03.027 18485877 PMC2728032

[177] Shibue T Weinberg RA. EMT, CSCs, and drug resistance: the mechanistic link and clinical implications. Nat Rev Clin Oncol. (2017) 14:611–29. 10.1038/nrclinonc.2017.44 28397828 PMC5720366

[178] Zhu Y Karakhanova S Huang X Deng SP Werner J Bazhin AV. Influence of interferon-α on the expression of the cancer stem cell markers in pancreatic carcinoma cells. Exp Cell Res. (2014) 324:146–56. 10.1016/j.yexcr.2014.03.020 24726912

[179] Clatot F Cornic M Berghian A Marchand V Choussy O El Ouakif F CXCL12 and CXCR4, but not CXCR7, are primarily expressed by the stroma in head and neck squamous cell carcinoma. Pathology. (2015) 47:45–50. 10.1097/PAT.0000000000000191 25474514

[180] Eckert F Schilbach K Klumpp L Bardoscia L Sezgin EC Schwab M. Potential role of CXCR4 targeting in the context of radiotherapy and immunotherapy of cancer. Front Immunol. (2018) 9:3018. 10.3389/fimmu.2018.03018 30622535 PMC6308162

[181] Katayama A Ogino T Bandoh N Nonaka S Harabuchi Y. Expression of CXCR4 and its down-regulation by IFN-γ in head and neck squamous cell carcinoma. Clin Cancer Res. (2005) 11:2937–46. 10.1158/1078-0432.CCR-04-1470 15837745

[182] Nagase H Miyamasu M Yamaguchi M Imanishi M Tsuno NH Matsushima K. Cytokine-mediated regulation of CXCR4 expression in human neutrophils. J Leukoc Biol. (2002) 71:711–7. Available online at: https://jlb.onlinelibrary.wiley.com/11927659

[183] Lollini PL De Giovanni C Del Re B Nicoletti G Prodi G Nanni P. Interferon-mediated enhancement of metastasis. Are MHC antigens involved? Clin Exp Metastasis. (1987) 5:277–87. 10.1007/BF00120723 3117468

[184] Zöller M Strubel A Hämmerling G Andrighetto G Raz A Ben-Ze'ev A. Interferon-gamma treatment of B16 melanoma cells: opposing effects for non-adaptive and adaptive immune defense and its reflection by metastatic spread. Int J Cancer. (1988) 41:256–66. 10.1002/ijc.2910410217 3123403

[185] McMillan TJ Rao J Everett CA Hart IR. Interferon-induced alterations in metastatic capacity, class-1 antigen expression and natural killer cell sensitivity of melanoma cells. Int J Cancer. (1987) 40:659–63. 10.1002/ijc.2910400515 2445702

[186] Ramani P Balkwill FR. Enhanced metastases of a mouse carcinoma after *in vitro* treatment with murine interferon gamma. Int J Cancer. (1987) 40:830–4. 10.1002/ijc.2910400621 3121523

[187] Xu Y-H Li Z-L Qiu S-F. IFN-γ induces gastric cancer cell proliferation and metastasis through upregulation of integrin β3-mediated NF-κB signaling. Transl Oncol. (2018) 11:182–92. 10.1016/j.tranon.2017.11.008 29306706 PMC5755748

[188] Mojic M Takeda K Hayakawa Y. The dark side of IFN-γ: its role in promoting cancer immunoevasion. Int J Mol Sci. (2017) 19:89. 10.3390/ijms19010089 29283429 PMC5796039

[189] Liu C Gao AC. IFNγ, a double-edged sword in cancer immunity and metastasis. Cancer Res. (2019) 79:1032–3. 10.1158/0008-5472.CAN-19-0083 30877097

[190] Liu Y Lv J Liu J Liang X Jin X Xie J. STAT3/p53 pathway activation disrupts IFN-β–induced dormancy in tumor-repopulating cells. J Clin Invest. (2018) 128:1057–73. 10.1172/JCI96329 29431732 PMC5824876

[191] Liu Y Liang X Yin X Lv J Tang K Ma J. Blockade of IDO-kynurenine-AhR metabolic circuitry abrogates IFN-γ-induced immunologic dormancy of tumor-repopulating cells. Nat Commun. (2017) 8:15207. 10.1038/ncomms15207 28488695 PMC5436221

[192] Farrar JD Katz KH Windsor J Thrush G Scheuermann RH Uhr JW. Cancer dormancy. VII. a regulatory role for CD8+ T cells and IFN-γ in establishing and maintaining the tumor-dormant state. J Immunol. (1999) 162:2842–9. 10072532

[193] Kmieciak M Payne KK Wang X-Y Manjili MH. IFN-γ Rα is a key determinant of CD8+ T cell-mediated tumor elimination or tumor escape and relapse in FVB mouse. PLoS ONE. (2013) 8:e82544. 10.1371/journal.pone.0082544 24324806 PMC3855782

[194] Essers MAG Offner S Blanco-Bose WE Waibler Z Kalinke U Duchosal MA. IFNα activates dormant haematopoietic stem cells *in vivo*. Nature. (2009) 458:904–8. 10.1038/nature07815 19212321

[195] Pietras EM Lakshminarasimhan R Techner J-M Fong S Flach J Binnewies M. Re-entry into quiescence protects hematopoietic stem cells from the killing effect of chronic exposure to type I interferons. J Exp Med. (2014) 211:245–62. 10.1084/jem.20131043 24493802 PMC3920566

[196] Dunn GP Koebel CM Schreiber RD. Interferons, immunity and cancer immunoediting. Nat Rev Immunol. (2006) 6:836–48. 10.1038/nri1961 17063185

[197] McCoach CE Bivona TG. The evolving understanding of immunoediting and the clinical impact of immune escape. J Thorac Dis. (2018) 10:1248–52. 10.21037/jtd.2018.03.60 29708132 PMC5906217

[198] Vahidian F Duijf PHG Safarzadeh E Derakhshani A Baghbanzadeh A Baradaran B Interactions between cancer stem cells, immune system and some environmental components: friends or foes? Immunol Lett. (2019) 208:19–29. 10.1016/j.imlet.2019.03.004 30862442

[199] Bhatia A Kumar Y. Cancer stem cells and tumor immunoediting: putting two and two together. Expert Rev Clin Immunol. (2016) 12:605–7. 10.1586/1744666X.2016.1159133 26919116

